# Characterization of a Ten-Cysteine Peptide from Scorpion That Inhibits Classical Complement Activation via C4/C4b

**DOI:** 10.3390/biom16101520

**Published:** 2026-10-19

**Authors:** Xudong Luo, Wen Zhu, Yuwei Wang, Xiaolin Deng, Zhiping Zhai, Wangmei Duan, Xiangdong Ye, Li Ding, Fang Sun, Hongyi Luo, Chenhu Qin, Zongyun Chen

**Affiliations:** 1 Hubei Key Laboratory of Embryonic Stem Cell Research and Hubei Key Laboratory of Wudang Local Chinese Medicine Research of Institute of Biomedicine, College of Basic Medicine, Hubei University of Medicine, Shiyan 442000, China; 2 Department of Clinical Laboratory, Dongfeng Hospital, Hubei University of Medicine, Shiyan 442008, China

**Keywords:** scorpion toxin, anticomplement peptide, ascaris-type, C4/C4b, ischemia–reperfusion injury

## Abstract

Scorpion toxin peptides are useful bioactive substance resources, but no anticomplement peptides have been reported from scorpions. Here, we attempt to explore scorpion-derived anticomplement peptides by scanning a small and specific scorpion toxin peptide library. Given that complement activation constitutes a protease-driven proteolytic cascade reaction, we focused on two kinds of serine protease inhibitor-like peptides from scorpion Kunitz-type and Ascaris-type toxin subfamilies. Assays of complement-mediated erythrocyte lysis showed that one scorpion peptide SjAPI-2, with an Ascaris-type structural fold inhibited classical complement activation in a concentration-dependent manner. Complement-deficient experiments revealed that the anti-complement effect of SjAPI-2 was markedly enhanced in sera reconstituted with deficient complement proteins C3 or C4, and suggested that C3 and C4 are associated with the anticomplement effect of SjAPI-2. Combined ELISA and Co-IP assays further revealed that SjAPI-2 can directly bind to C4 and C4b. These results indicated that SjAPI-2 can inhibit classical complement activation via binding to C4 and C4b, which blocks the upstream proteolytic cleavage of C4 as well as the downstream cleavage and activation of C3. Alanine-scanning mutagenesis showed that SjAPI-2 suppressed complement activation via its functional loop from K30 to V38. Myocardial ischemia–reperfusion injury experiment showed that SjAPI-2 limited the extent of myocardial infarction and exerted a cardioprotective effect in vivo. To the best of our knowledge, SjAPI-2 is the first anticomplement toxin peptide from a scorpion, suggesting that scorpion venom peptides might be a new resource for anticomplement peptide discovery. The unique ten-cysteine peptide scaffold of SjAPI-2 also provided a novel lead drug template for C4/C4b associated complement diseases.

## 1. Introduction

Scorpions are one of the most ancient arthropods and have survived on Earth for over 400 million years. In China, scorpions were used as an important medicinal material for anticonvulsant, antiepileptic, analgesic, anti-inflammatory, antitumor, anticoagulant and microcirculation-improving effects [1,2,3,4]. Despite the molecular diversity of scorpions’ disulfide-bridged peptide toxins [5], only four major structural scaffolds have been found: (1) the classical CSα/β fold, which comprises one or two short α-helices connected to a triple-stranded anti-parallel β-sheet stabilized by three or four disulfide bonds [6]; (2) the other is the unique cystine-stabilized α-helix-loop helix (CSα/α) fold, which comprises two α-helices [7]; (3) the Kunitz-type fold, which is a cysteine-rich peptide chain with alpha and beta fold, stabilized by three conserved disulfide bridges [8]; and (4) the Ascaris-type fold, which have a common structural characteristic with four short β-strands arranged in two approximately perpendicular β-sheets and stabilized by five disulfide bridges: Cys I–Cys VII, Cys II–Cys VI, Cys III–Cys V, Cys IV–Cys X, and Cys VIII–Cys IX [9,10,11]. Scorpion toxins represent an important resource for the discovery of peptide drugs and exhibit diverse biological functions [12,13,14,15,16], including serine protease inhibitory activity, potassium channel inhibitory activity, sodium channel regulatory activity, analgesic activity, antitumor activity, as well as antibacterial and antiviral activities, but no anticomplement scorpion toxin peptides have been reported [17].

The complement system is a key immune mediator machine for defense against infection and an important driver of inflammation. However, inappropriate or uncontrolled activation of complement can cause local and/or systemic inflammation, tissue damage and disease [18,19]. Anti-complement therapies have been used as a new strategy for many complement-associated diseases that include infectious, inflammatory, degenerative, traumatic and neoplastic disorders [20,21,22]. Based on molecular nature, anti-complement drugs could be divided into three major categories: nucleic acid-based drugs, small-molecule drugs and monoclonal antibodies, protein biologics and peptides [23]. Nucleic acid-based drugs include ARC1905 that is a C5 aptamer [24], ALN-CC5 that is a C5 RNA interference (RNAi) molecule [25], and Spiegelmers NOX-D19 to NOX-D21 targeting C5a [26]. Small-molecule inhibitors include AVACOPAN and DF2593A targeting C5aR [27,28], Compstatin, Cp40, APL-1 and APL-2 targeting C3 [29]. Monoclonal antibody drugs include TNT003 and TNT009 targeting complement fragment C1s [30], Narsoplimab targeting MBL-associated serine protease 2 (MASP2) [31], Lampalizumab targeting Factor D [32], IFX-1 targeting C5a [33], and eculizumab, LFG316, ALXN1210 and ALXN550 targeting C5 [34]. Protein biologic drugs include Berinert, Ruconest and Cinryze targeting C1; AMY-201 that is a mini FH [35]; TT30 that is a CR2–FH hybrid [36]; and TP10 that is a soluble form of complement receptor type 1 (CR1) [37]. Peptide drugs include macrocyclic peptide RA101348 targeting C5 [38], Compstatin and its analog Pegcetacoplan targeting C3 [29], zilucoplan targeting C5 [39], and a cyclic peptide targeting C5aR1 [40]. Scorpion venom is predominantly composed of bioactive scorpion toxin peptides, and the present work focuses on the identification of complement-inhibitory peptides, with particular attention paid to the anti-complement functions of disulfide-rich cyclic peptides [3].

During complement activation, serine proteases play important roles in the initiation, amplification and effector processes, suggesting that serine protease inhibitors might be a new source of anticomplement-targeting lead drugs [41]. Among the four folds of scorpion toxins, the Kunitz-type fold and the Ascaris-type fold have been reported to have potential serine protease inhibitory activity [9]. Here, based on a classical pathway anti-complement activity experimental platform, we screened recombinant Kunitz-type and Ascaris-type scorpion toxin peptides stored in our laboratory that we had prepared before, including 5 Kunitz-type scorpion toxins, 3 Ascaris-type scorpion toxins, and the CSα/β scorpion toxin BmαTX14 and the Kunitz-type snake toxin BF9 as control peptides. Two scorpion venom-derived Ascaris-type peptides, SjAPI-2 and BmHDP, were found to have apparent anti-complement activity, and SjAPI-2 showed the best anti-complement activity. Our work highlights the functional significance of natural peptide resources for anticomplement drug discovery and provides a new clue for new non-antibody scaffold discovery for complement-associated diseases.

## 2. Materials and Methods

### 2.1. Peptides and Complement Protein

All the recombinant scorpion toxin peptides that were used in this paper have been reported in a previous work, and were stored in our laboratory in dry powder form at −80 °C, including scorpion toxin peptides BmKTT-1, BmKTT-2, LmKTT-1a, Hg1 [42], SdPI [43], SjAPI [11], SjAPI-2 [9], BmHDP [44], BmαTX14 [45], snake toxin peptide BF9 [46], and scorpion toxin peptide SjAPI-2 mutants K30A, H31A, C32A, P33A, Q34A, G35I, S35A, C36A, F37A, and V38A. Purified human complement proteins C1, C1q, C2, C3, C3b, C4, C4b, C5, and C6 were purchased from Complement Technology, Inc. (Tyler, TX, USA).

### 2.2. Classical Pathway Hemolytic Assays

Equal volumes of normal human serum (NHS) at the critical concentration and 10 μM peptide solution were mixed and incubated in a 37 °C water bath for 10 min. EA (Erythrocyte–Antibody)-sensitized erythrocytes and GVB (Gelatin Veronal Buffer) were then added, followed by another 30 min incubation at 37 °C. The reaction mixtures were centrifuged at 5000 rpm for 10 min at 4 °C. A 200 μL aliquot of supernatant from each tube was transferred to a 96-well plate, and absorbance was measured at 405 nm. Data were plotted with peptide treatment on the x-axis and hemolysis inhibition rate on the y-axis. Complement group, complete lysis group and GVB blank group were set up as parallel controls in the assay. The complement inhibition rate of the peptide was calculated using the formula: Complement inhibition rate (%) = (A_405control group_ − A_405peptide group_)/(A_405control group_ − A_405 GVB group_) × 100%. Equal volumes of critical-concentration NHS and peptide solutions at gradient concentrations were mixed and incubated at 37 °C for 10 min in a water bath. EA and GVB buffer were added, and the mixtures were incubated for another 30 min at 37 °C. After centrifugation at 5000 rpm and 4 °C for 10 min, 200 μL supernatant from each tube was transferred to a 96-well plate for absorbance detection at 405 nm. Hemolysis inhibition rate was plotted against peptide concentration on the x-axis. NHS, serially diluted at ratios of 1:20, 1:30 and 1:40, was separately mixed with an equal volume of 10 μM peptide solution, followed by 10 min incubation in a 37 °C water bath. EA and GVB buffer were added, and the samples were incubated for 30 min at 37 °C. After centrifugation at 5000 rpm for 10 min at 4 °C, 200 μL supernatant per tube was loaded into a 96-well plate, and absorbance was recorded at 405 nm. Hemolysis inhibition rate was plotted on the y-axis against serum dilution conditions on the x-axis. Complement group, complete lysis group and GVB group were included as controls for this experiment.

### 2.3. Complement-Depleted Serum Experiments

Equal volumes of 1:20 diluted normal human serum (NHS) and peptide at the critical concentration were mixed and incubated in a 37 °C water bath for 10 min. Then 100 μL of complement-depleted serum and 100 μL of EA (antibody-sensitized erythrocytes) were added, followed by incubation at 37 °C for another 30 min. The mixture was centrifuged at 5000 rpm for 10 min at 4 °C. A 200 μL aliquot of the supernatant was collected, and its absorbance was measured at 405 nm. The experimental groups included the peptide group, complement group, depleted serum group, depletion control group, non-depleted serum group, non-depleted serum control group, GVB buffer group, and complete lysis group.

### 2.4. ELISA

Wells of a 96-well plate were coated overnight at 4 °C with 100 μL per well of coating diluent containing human complement components C1, C1q, C2, C3, C3b, C4, C4b, C5 and C6 at 4 μg/mL, BSA at 4 μg/mL serving as the negative control, and coating buffer without any complement component serving as the blank control. After three washes with PBST buffer, the plate was blocked with PBS containing 2% BSA at 37 °C for 1 h. Following blocking and another three PBST washes, serially diluted test peptides prepared in peptide diluent at concentrations of 0, 5, 10, 20 and 40 μg/mL were added at 100 μL per well, and the plate was incubated at 37 °C for 1 h.

The plate was washed three times with PBST, then 100 μL of anti-6×His monoclonal antibody diluted 1:1000 was added as the primary antibody, followed by incubation at 37 °C for 1 h. After three PBST washes, 100 μL of HRP-conjugated goat anti-mouse IgG diluted 1:5000 was added as the secondary antibody and incubated at 37 °C for 1 h. The plate was rinsed three times with PBST before the addition of 50 μL TMB substrate for color development in the dark at room temperature for 20 min. The reaction was terminated by adding 50 μL of 2 M H_2_SO_4_, and absorbance values were read at 450 nm using a microplate reader.

### 2.5. Co-IP

Four hundred microliters of fully resuspended beads were transferred into an EP tube, mixed with 1 mL ice-cold TBS by gentle inversion, centrifuged at 4000 rpm for 5 min at 4 °C, and the supernatant was discarded. This washing step was repeated twice. The resulting bead pellet (approximately 100 μL) was resuspended in an equal volume of ice-cold TBS to prepare a 50% bead slurry. Three EP tubes were labeled A, B and C, with 40 μL of the bead suspension added to each tube. Tubes A and B received 20 μL peptide solution (1 mg/mL) together with 1 μL mouse anti-6×His monoclonal antibody from Proteintech Group, Inc. (Wuhan, China), while tube C received only 1 μL mouse anti-6×His monoclonal antibody. The total volume in each tube was adjusted to 500 μL using ice-cold TBS, followed by rotational incubation at 4 °C for 4 h. After incubation, the tubes were centrifuged at 4000 rpm for 5 min at 4 °C, and the supernatant was removed. The beads were resuspended in 500 μL ice-cold TBST, centrifuged again under the same conditions, and the supernatant was discarded; this TBST wash was performed once more. The beads were then resuspended in 500 μL ice-cold TBS, centrifuged at 4000 rpm for 5 min at 4 °C, and the supernatant was discarded. Each tube was supplemented with 10 μg of the target complement component, and the total volume was brought up to 500 μL with ice-cold TBS. The mixtures were incubated overnight with gentle rotation at 4 °C. Upon completion of incubation, samples were centrifuged at 4000 rpm for 5 min at 4 °C, and the supernatant was aspirated. The beads were washed twice with ice-cold TBST as described above, followed by one final wash with ice-cold TBS using identical centrifugation parameters, with supernatant discarded each time. The final bead pellet (approximately 20 μL) was mixed with an equal volume of 2× protein loading buffer, and the mixture was boiled in a water bath for 10 min. At last, all the samples were tested by Western blotting.

### 2.6. Establishment of Myocardial Ischemia/Reperfusion Injury Model in C57BL/6 Mice

Fifteen male C57BL/6 mice weighing about 25 g were obtained from the Animal Experiment Center of Hubei University of Medicine and housed under sterile conditions. Animals were randomly divided into three groups: 3 mice in the sham operation group (SO), 6 mice in the ischemia/reperfusion injury group (I/RI), and 6 mice in the peptide treatment group. All reagents or drugs were administered via tail vein injection. To achieve a target circulating concentration of 10 μM for peptide SjAPI-2 in C57BL/6 mice, the peptide injection dose was set at 5.36 mg/kg. For 25 g mice, the total blood volume (TBV) was calculated as 1.8 mL, and values for mice of other body weights were extrapolated proportionally. The chest fur of mice was depilated one day in advance. Mice were anesthetized via inhalation of 1.5–2% isoflurane and fixed with adhesive tape. The skin directly above the heart was incised, and a purse-string suture was pre-placed on the chest skin using 4-0 sutures with attached needles. The pectoralis major and pectoralis minor muscles were bluntly dissected with ophthalmic forceps. A mosquito hemostat was used to pierce the fourth intercostal space to exteriorize the heart. A loose knot was tied around the left anterior descending coronary artery (LAD) at a site 3 mm below the left atrial appendage, with an approximately 8 mm suture end reserved. The heart was quickly returned to the thoracic cavity. The chest cavity was closed with air expelled, followed by tightening the purse-string suture. Erythromycin eye ointment was applied to disinfect the surgical wound, and mice were placed on a heating blanket until recovery from anesthesia. Sham operation group (SO): suture was threaded under the coronary artery without ligation; normal saline was administered via tail vein injection before ligation. I/R injury (I/RI) group: the left anterior descending coronary artery was ligated, and normal saline was injected through the tail vein prior to ligation. Peptide treatment group: the left anterior descending coronary artery was ligated, and peptide diluted with normal saline was injected via the tail vein before ligation.

### 2.7. Evans Blue/TTC Staining

At the end of reperfusion, mice were re-anesthetized with inhaled isoflurane. The diaphragm and bilateral ribs were cut open, and the sternum was flipped upward and fixed to fully expose the heart. The pericardium was carefully removed, and the LAD was re-ligated using 6-0 needle-attached sutures. The aorta was isolated and clamped with forceps (care was taken to avoid aortic rupture). A volume of 0.1–0.2 mL of 0.5% Evans Blue (diluted in normal saline) was injected retrogradely, and the heart was observed for 1 min after injection. After successful dye perfusion, the heart was excised, rinsed to flush out intracardiac blood, placed on a Petri dish lid and covered with a drop of water, then frozen at −20 °C for 30 min. The frozen heart was cut into 1 mm-thick transverse sections perpendicular to the long cardiac axis. Sections were transferred into a 6-well plate, immersed in 2 mL of 0.5% TTC solution (prepared with PBS), and incubated with shaking at 37 °C for 30 min in the dark. The TTC solution was aspirated off, and the slices were rinsed three times with distilled water by gentle pipetting until the washing solution became clear. After complete removal of waste liquid, tissues were fixed in 4% paraformaldehyde for 30 min. Images were captured, and the infarct size was quantified using the microscopic imaging software Phmias2008 Ver3.0. Calculation of myocardial infarct percentage (MI%): For each cardiac slice, MI% = area of infarcted region/total area of the slice.

### 2.8. Statistical Analysis

All experimental data are expressed as mean ± standard deviation. Unpaired Student’s *t*-test was adopted for statistical analysis. ns indicates *p* > 0.05, * indicates *p* < 0.05, ** indicates *p* < 0.01, and *** indicates *p* < 0.001.

## 3. Results

### 3.1. Scorpion-Derived Ascaris-Type Peptide SjAPI-2 Is a New Anticomplement Agent

**Figure 1:**
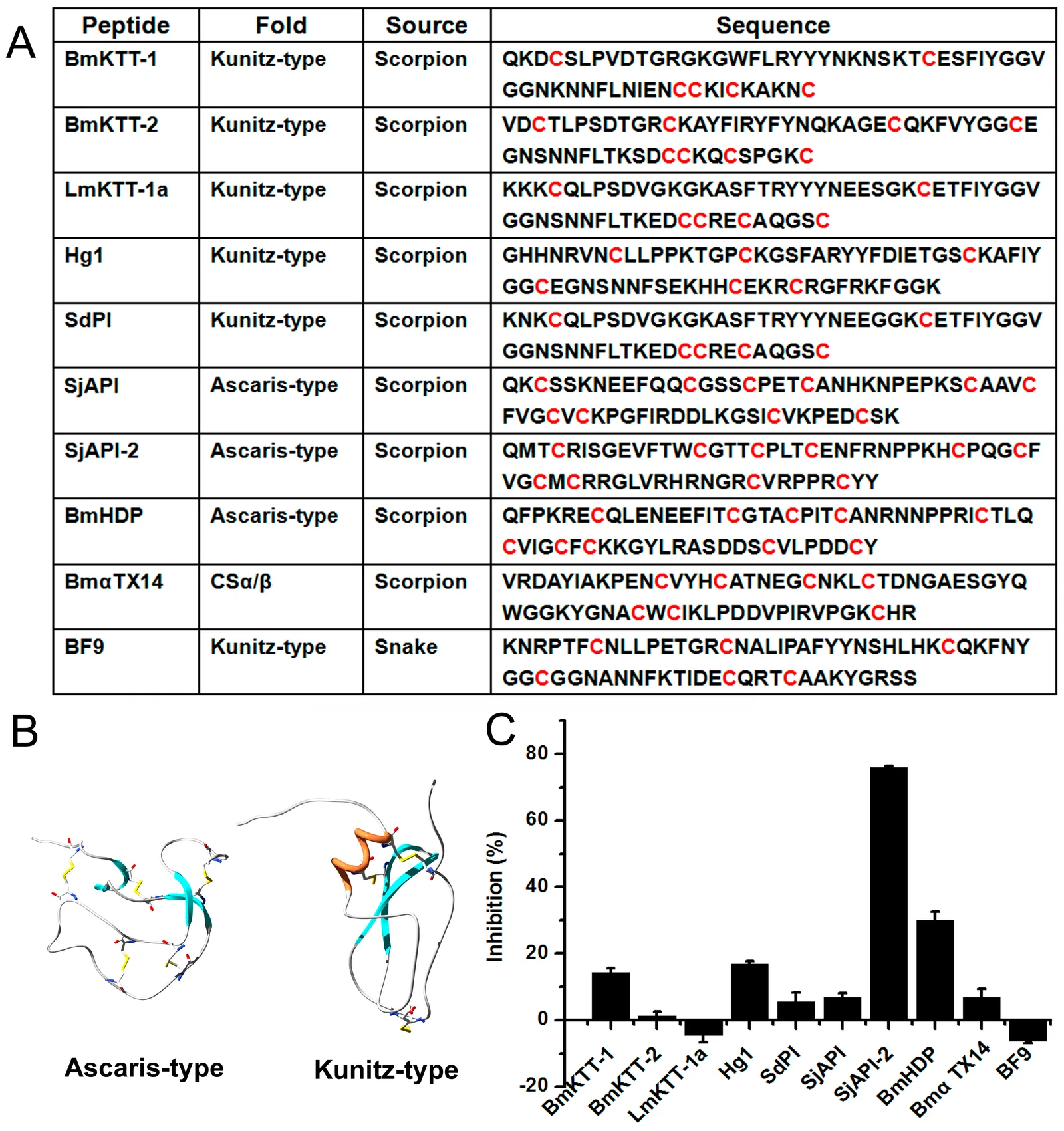
Scanning scorpion-derived anticomplement peptides from Kunitz-type and Ascaris-type toxin peptide subfamilies. (**A**). Nine scorpion toxin peptides with Kunitz-type and Ascaris-type structural folds. BmKTT-1, BmKTT-2, BmHDP and BmαTX14 from the scorpion *Buthus martensii Karsch*; LmKTT-1a and SdPI from the scorpion *Lychas mucronatus*; Hg1 from the scorpion *Heterometrus fulvipes*; SjAPI and SjAPI-2 from the scorpion *Scorpiops jendeki*; and the control peptide BF9 from the snake *Bungarus fasciatus*. Cysteine residues are marked in red. (**B**). 3-D structures of two representative scorpion toxin peptides with Kunitz-type and Ascaris-type structural folds, respectively. (**C**). Anticomplement activity scanning towards the classical complement pathway of scorpion toxin peptides.

**Figure 2:**
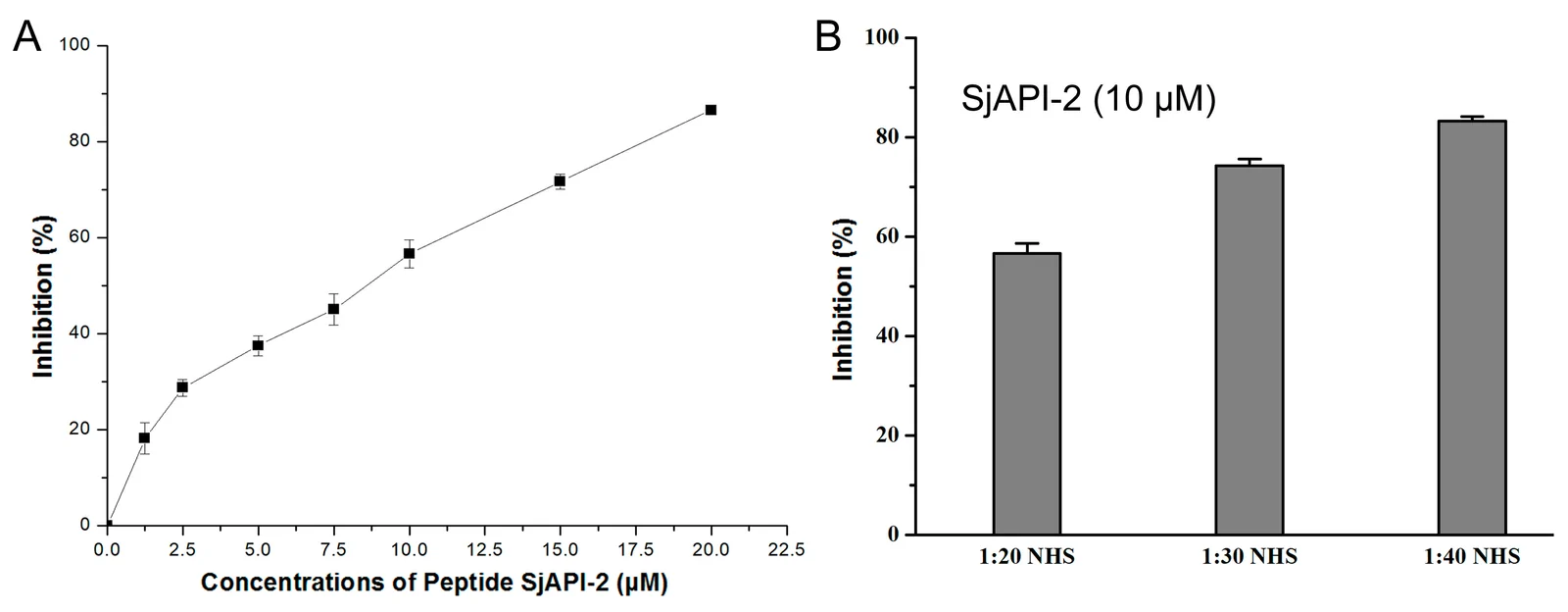
The anticomplement effect of SjAPI-2 exhibited obvious concentration-dependent characteristics. (**A**). The anticomplement effect of peptide SjAPI-2. (**B**). The anticomplement effect of normal human serum.

Our laboratory has previously constructed a small library of Kunitz-type and Ascaris-type peptides derived from scorpions, and identified abundant serine protease inhibitors among these scorpion bioactive peptides [8,42,47]. Using the scorpion bioactive peptide resource library established in our laboratory, we screened 9 Kunitz-type and Ascaris-type peptides for their anticomplement activity against the complement classical pathway, and snake Kunitz-type toxin BF9 as a negative control. The tested peptides were BmKTT-1, BmKTT-2, LmKTT-1a, Hg1, SdPI, SjAPI, SjAPI-2, BmHDP, BmαTX14 and BF9, with a final concentration of 10 μM for each peptide in the reaction system (Figure 1). The results demonstrated that the Ascaris-type peptide SjAPI-2 exhibited the strongest complement inhibitory effect, achieving a hemolysis inhibition rate of 75% at the peptide concentration of 10 μM. We therefore further evaluated the complement inhibitory activity of SjAPI-2 at different concentrations and found that its anticomplement activity exhibited favorable concentration dependence. To further verify the anticomplement activity of SjAPI-2, we also examined the inhibitory effect of SjAPI-2 on complement at varying serum concentrations, and the results revealed that the complement inhibitory activity of SjAPI-2 was also serum-concentration dependent, indicating that the peptide reduced erythrocyte lysis by suppressing complement activation (Figure 2).

### 3.2. Scorpion Peptide SjAPI-2 Inhibits Complement Activation via C4/C4b Binding

**Figure 3:**
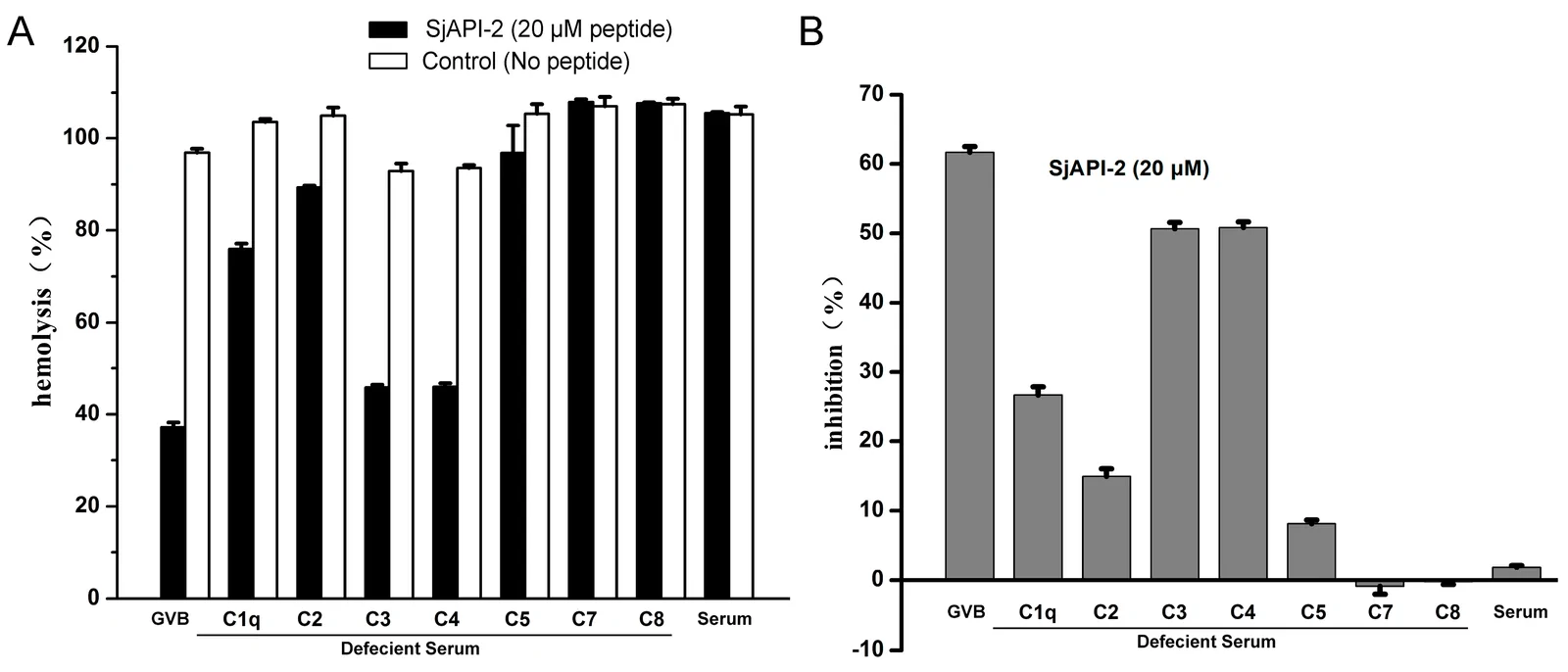
The complement-depletion assay indicated that potential anticomplement targets of the peptide might be C3 and C4. (**A**). Hemolysis rate of sensitized sheep erythrocytes upon reconstitution with deficient sera after incubation of peptide SjAPI-2 with complement. (**B**). Inhibition rate of peptide SjAPI-2 against groups reconstituted with different complement-component-deficient sera.

Complement was mixed with the peptide at its critical concentration, followed by the addition of individual complement components for reconstitution. The reconstituted components included C1q, C2, C3, C4, C5, C7 and C8, and absorbance was measured at 405 nm. Experimental groups were set up as the peptide treatment group, complete lysis group, complement group, complement-depleted serum group and depletion control group. If the peptide targeted the depleted complement component, no hemolysis would occur in the reaction system; otherwise, hemolysis would take place. Results from the complement component depletion assay demonstrated that the main molecular targets responsible for the anticomplement activity of SjAPI-2 were complement proteins C3 and C4 (Figure 3).

**Figure 4:**
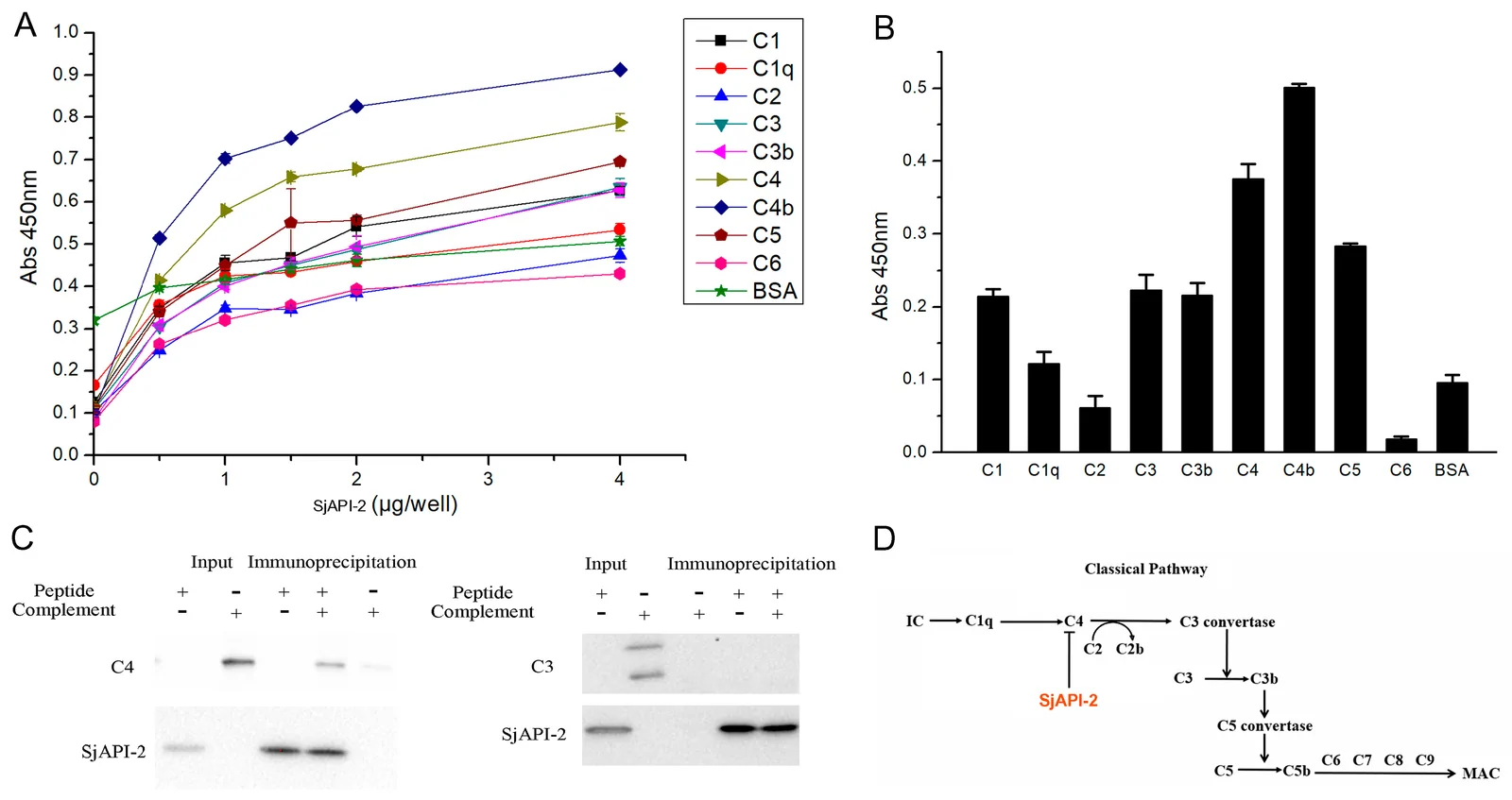
ELISA and Co-IP assays indicated that the major binding proteins of SjAPI-2 were C4 and C4b. (**A**). Binding assay of peptide SjAPI-2 to distinct complement proteins at varying concentrations. (**B**). Binding assay of peptide SjAPI-2 to distinct complement proteins at 4 μg. (**C**). Co-IP of peptide SjAPI-2 and complement protein C4 (western blot original can be found at Figure S2). (**D**). Schematic of the anticomplement action of SjAPI-2 that inhibits the classical complement pathway via binding to C4 and C4b.

ELISA plate wells were coated with antibodies against human complement proteins C1q, C2, C3, C4, C5, C6, C7, C8 and C9 for 12 h. After washing, the wells were blocked with PBS containing 2% bovine serum albumin (BSA) at 37 °C for 2 h. The wells were washed again, and horseradish peroxidase (HRP)-conjugated anti-His monoclonal antibody was added, followed by 1 h incubation at 37 °C. Unbound HRP-labeled antibody was removed by washing, then TMB substrate solution was added to trigger the colorimetric reaction catalyzed by the enzyme. Results from the ELISA binding assay demonstrated that SjAPI-2 predominantly bound to complement proteins C4/C4b (Figure 4).

ELISA results showed that peptide SjAPI-2 exhibited substantial binding to complement C4, suggesting favorable specificity, whereas its binding to other complement proteins such as C3 was weak and likely non-specific. Therefore, it was necessary to further perform Co-IP experiments for validation. Based on the above results from complement depletion assays and ELISA binding tests, complement proteins C3 and C4 exhibited prominent responses. We therefore further verified the binding interactions between SjAPI-2 and complement C3/C4 via co-immunoprecipitation (Co-IP). Western blot results showed that complement C4, but not C3, could be pulled down together with SjAPI-2 using an anti-His monoclonal antibody, whereas free C4 alone could not be precipitated (Figure 4). This finding confirmed the specific binding between C4 and the SjAPI-2 peptide.

Although C3-deficient and C4-deficient sera exhibited comparably strong inhibitory effects in the complement-depletion assay, ELISA and Co-IP experiments further confirmed that peptide SjAPI-2 majorly binds to complement C4 but not complement C3. Because the complement-depletion assay is a functional assay, binding of SjAPI-2 to complement C4 suppresses the activity of C3 convertase and thereby inhibits complement activation. Correspondingly, depletion of C3, the substrate of C3 convertase, in serum also attenuates complement activation, which explains why C3-deficient serum also displayed prominent effects in the complement depletion assay (Figure 4D). Taken together, complement component C4 is identified as the major anticomplement target of the scorpion toxin peptide SjAPI-2.

### 3.3. Structural–Activity Relationship of Anticomplement Peptide SjAPI-2

**Figure 5:**
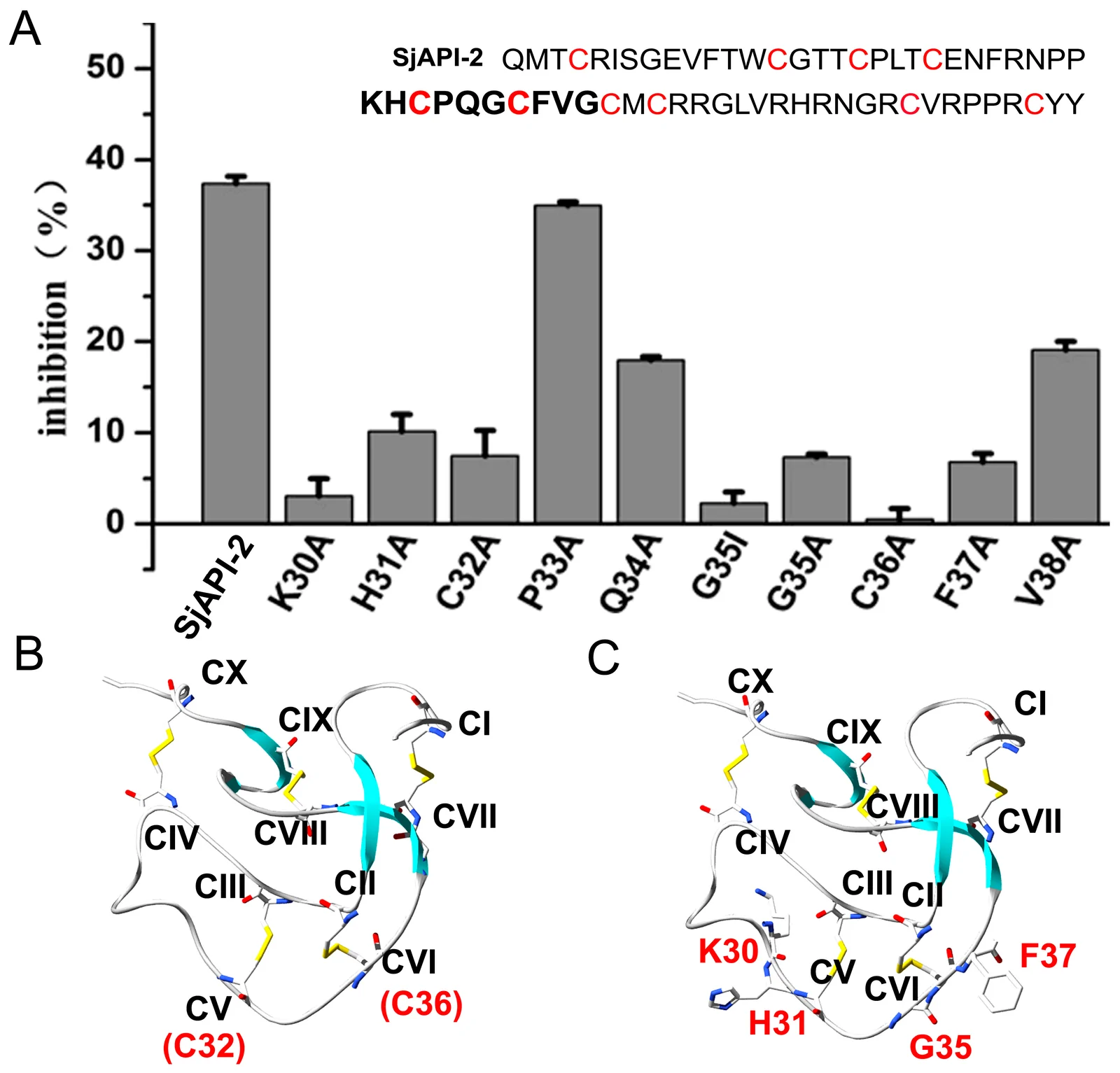
Structural and functional relationship of the anticomplement Ascaris-type peptide SjAPI-2. (**A**). Inhibition rate of classical-pathway anticomplement hemolysis by SjAPI-2 and its mutants. Cysteine residues are marked in red. (**B**). Disulfide-bond pairing pattern of SjAPI-2. C32 and C36 are marked in red. (**C**). Potential key residues of SjAPI-2 that inhibited the classical complement pathway and are marked in red.

It is well known that Ascaris-type peptides constitute an important class of serine protease inhibitors and have a common structural characteristic with four short β-strands arranged in two approximately perpendicular β-sheets and stabilized by five disulfide bridges: Cys I–Cys VII, Cys II–Cys VI, Cys III–Cys V, Cys IV–Cys X, and Cys VIII–Cys IX. The reactive site loop of Ascaris-type peptides is almost bounded by two disulfide bridges (Cys II–Cys VI and Cys III–Cys V) and is part of the long loop connecting strands β1 and β2 [47]. The loop region of SjAPI-2 spans residues C32 to C36. Accordingly, we performed alanine scanning mutagenesis on this region of SjAPI-2 by mutating amino acids at positions 30–38 to alanine (Ala), and investigated how these substitutions affected the anticomplement function of SjAPI-2. Given that glycine is a unique amino acid lacking a side chain, we designed two amino acid substitutions to confirm the experimental results. Then, we produced ten SjAPI-2 mutants following the same protocol used for the wild-type SjAPI-2 peptide: SjAPI-2K30A, SjAPI-2H31A, SjAPI-2C32A, SjAPI-2P33A, SjAPI-2Q34A, SjAPI-2G35A, SjAPI-2G35I, SjAPI-2C36A, SjAPI-2F37A and SjAPI-2V38A as we have described before [9,11]. The complement classical pathway hemolytic assay using sensitized sheep erythrocytes was employed to test whether these mutant peptides retained anticomplement activity, with all peptides tested at a final concentration of 10 μM (Figure 5). The results showed that mutations within the loop region led to varying degrees of reduction in the anticomplement activity of SjAPI-2. These findings indicate that the loop segment covering residues 30–38 is the key functional region responsible for the anticomplement effect of SjAPI-2.

### 3.4. Protective Role of Ischemia/Reperfusion Injury

**Figure 6:**
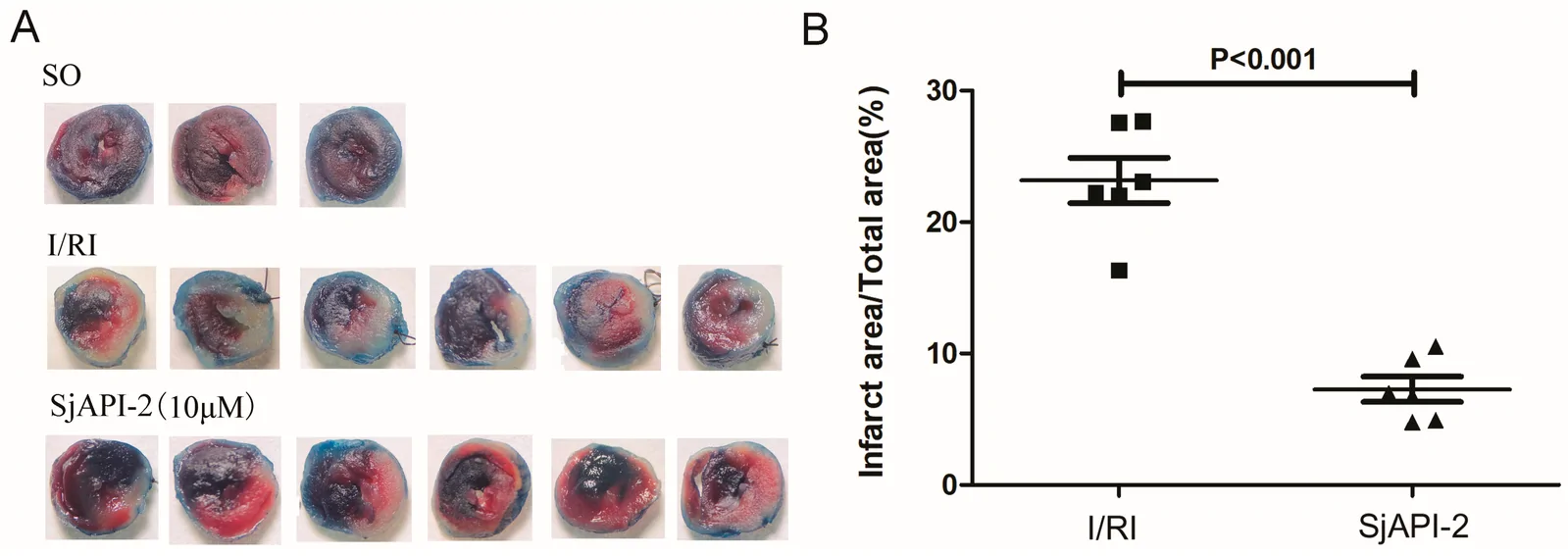
The protective effect of SjAPI-2 against myocardial ischemia–reperfusion injury. (**A**). Evans Blue/TTC staining of mouse cardiac tissues. White indicates the necrotic area, red denotes the ischemic area, and blue represents the non-risk area. (**B**). Effect of the peptide on the necrotic area size in mouse myocardium.

Ischemia/reperfusion injury is closely correlated with abnormal complement activation [48,49]. Evans Blue combined with TTC staining labels the non-risk area of mouse myocardium blue and the viable area red. The infarcted region lacks dehydrogenase activity and thus remains unstained, appearing white. The white infarct area in the peptide-treated group was smaller than that in the ischemia/reperfusion injury (I/RI) group, and the blue and red staining was also more distinct in the peptide group compared with the I/RI group. The ratio of infarct area to total ventricular area was calculated. Quantitative analysis showed that the myocardial infarct size in the I/RI group was significantly larger than that in the sham operation group (*p* < 0.0001). Compared with the I/RI group, administration of peptide SjAPI-2 markedly reduced the myocardial infarct size (Figure 6). These results suggest that SjAPI-2 can limit the extent of myocardial infarction and exert a cardioprotective effect against myocardial ischemia–reperfusion injury.

## 4. Discussion

Previous studies have demonstrated that scorpion venom is capable of directly activating the complement system, driving C3 cleavage and robust production of the chemoattractant anaphylatoxins C3a and C5a [50,51,52,53], which is similar to snake venom protein CVF [17]. Complement activation serves as a key driver of the inflammatory storm following scorpion envenomation. Notably, complement activation is far more pronounced in liver tissue than in other organs, accompanied by markedly elevated hepatic expression of IL-1β and TNF-α. Sole administration of scorpion toxins AahI and AahII is sufficient to activate the complement system [54]. The underlying mechanism likely involves targeting voltage-gated sodium channels on cell membranes, which triggers robust depolarization of neurons and immune cells accompanied by massive release of neurotransmitters and cytokines and drives explosive activation of the complement system in vivo. Scorpion toxin peptides AahI and AahII did not directly bind to complement components such as C3, C4 or others, which was different from snake venom protein CVF [17,54]. However, research on complement inhibition by scorpion toxin peptides remains largely unexplored to date [17]. In the present study, we constructed a small peptide library consisting of Kunitz-type and Ascaris-type scorpion peptides and performed functional screening assays. We identified a monomeric scorpion toxin peptide, SjAPI-2, that can directly bind complement proteins C4/C4b, thereby inhibiting activation of the complement classical pathway. This finding is of great significance for deepening our understanding of the biological functions of scorpion toxin peptides and facilitating the discovery of lead drugs derived from scorpion toxin peptides.

Within this type of peptide, the ten intramolecular cysteine residues form five disulfide bonds following the pairing patterns CI–CVII, CII–CVI, CIII–CV, CIV–CX, and CVIII–CIX. A loop structure is formed between residues CV and CIV [55]. SjAPI-2 is an Ascaris-type serine protease inhibitor composed of 62 amino acids with ten intramolecular cysteine residues from the scorpion *Scorpiops jendeki* [9,56,57]. Its five disulfide bonds are paired as C4–C40, C14–C36, C18–C32, C22–C60, and C42–C54, which are similar to those of its homologous scorpion-toxin peptides from the scorpion *Hadogenes troglodyte* (Figure S1) [58]. Our present work showed that SjAPI-2 inhibited complement activation by the classical functional loop region, and highlights a novel anticomplement peptide scaffold with ten cysteines.

Focusing on C4b, the key effector of the complement classical pathway, nanobodies combined with phage display screening technology have been used to develop a panel of nanobodies with varying binding affinities [59,60]. Among these candidates, hC4Nb8 stands out as a high-affinity nanobody generated by immunizing llamas with human C4b followed by phage display selection, achieving picomolar binding affinity. This nanobody specifically recognizes a structural epitope that becomes exposed exclusively upon C4 activation, thus binding only the activated form of C4b. Our current findings reveal that the major binding target of the scorpion toxin peptide SjAPI-2 is C4b, and this peptide also exhibits moderate binding affinity for C4 protein. This suggests that SjAPI-2 exerts dual effects on inhibiting the classical complement activation pathway via C4/C4b. First, it specifically recognizes structural regions exposed only after C4 activation, selectively targeting activated C4b and blocking the assembly of core convertases of the classical and lectin pathways by occupying critical binding interfaces. Second, it directly binds to complement C4 in its inactive native state. This property allows the peptide not only to block downstream cascade reactions triggered after C4 cleavage but also to prevent proteolytic cleavage of C4 by upstream C1s and MASP-2, thereby reducing the conversion of C4 to C4b at the source and inhibiting the initiation of the classical and lectin pathways at an earlier stage. Among existing camelid-derived nanobodies known to bind native C4, NbG3 and NbE11 show only limited inhibitory potency. NbG3 produces robust inhibition only under conditions of low serum complement concentration, while NbE11 exhibits even weaker inhibitory activity. At present, there are no high-affinity lead drug candidates that can potently bind resting native C4 and block its proteolytic cleavage. Given the SjAPI-2 C4-binding property, developing lead peptides with high affinity toward C4 represents a promising direction for future research of SjAPI-2 [61,62].

## 5. Conclusions

We found the first anticomplement peptide SjAPI-2 from a scorpion and provide a novel ten-cysteine peptide scaffold for C4/C4b targeting complement drug discovery.

## Data Availability

The original contributions presented in this study are included in the article/Supplementary Material. Further inquiries can be directed to the corresponding author(s).

## Supplementary Materials

The following supporting information can be downloaded at: https://www.mdpi.com/article/10.3390/biom16101520/s1, Figure S1: SjPAI-2 and its homologous peptides HtPi1 and HtPi2 with ten cysteines. A. Sequence alignments of SjPAI-2 and its homologous peptides HtPi1 and HtPi2. B. Structural modeling and analyses of SjAPI-2; Figure S2: Western Blot Original Image.
