# Supplementary material for: Characterization of a Ten-Cysteine Peptide from Scorpion That Inhibits Classical Complement Activation via C4/C4b
Source: Biomolecules. 2026 Oct 19;16(10):1520. doi: 10.3390/biom16101520 (PMC13702167; doi:10.3390/biom16101520)
Supplement: Supplementary file 1 [file biomolecules-16-01520-s001.zip › biomolecules-4530224-supplementary.pdf]

Supplementary

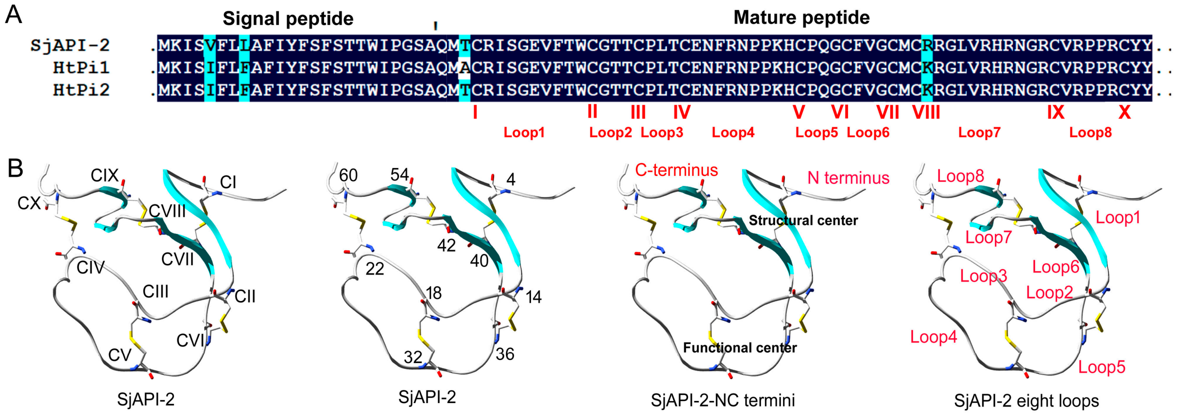

Figure S1. SjPAI-2 and its homologous peptides HtPi1 and HtPi2 with ten cysteines. A. Sequence alignments of SjPAI-2 and its homologous peptides HtPi1 and HtPi2. B. Structural modeling and analyses of SjAPI-2.

Figure S2. Western Blot Original Image.

(A) SjAPI-2 and C3

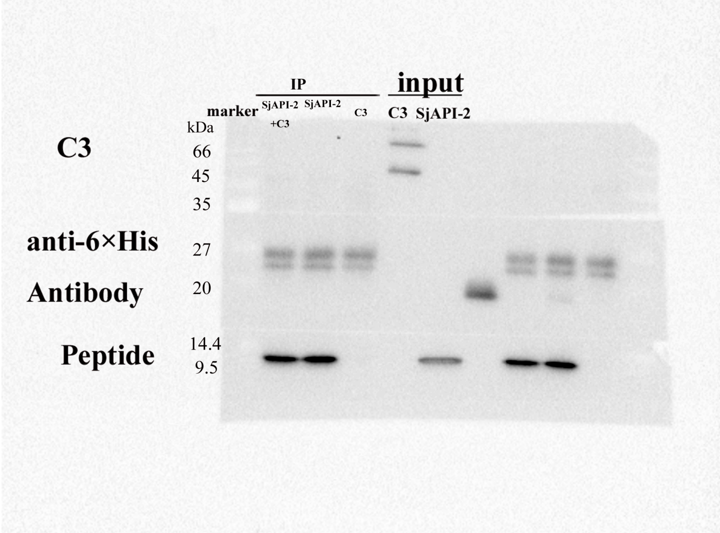

(B) SjAPI-2 and C4 (Long-time exposure to facilitate visualization of SjAPI-2 peptide)

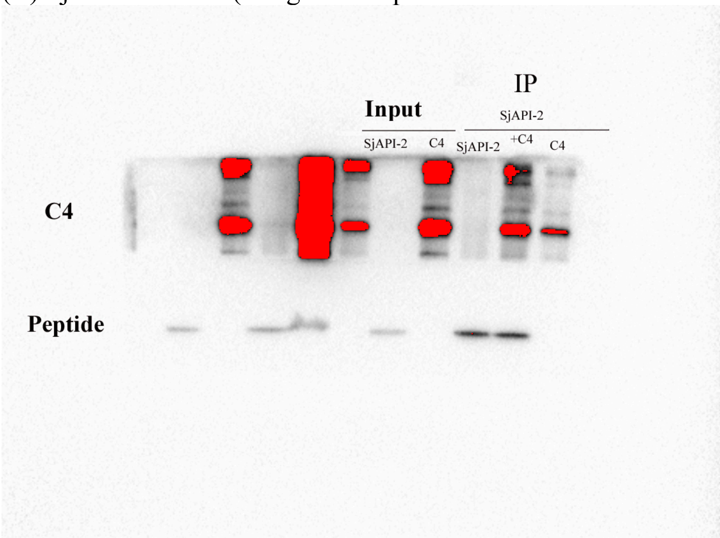

(C) SjAPI-2 and C4 (short-time exposure to facilitate visualization of C4)

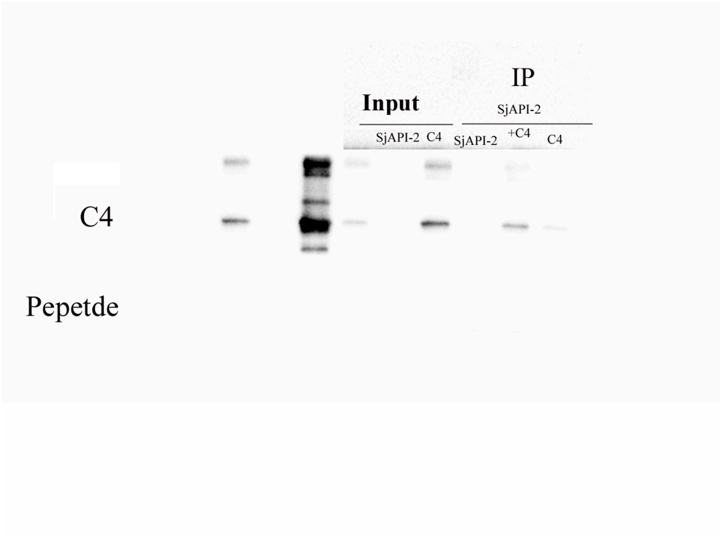
IP
Input
SjAPI-2
SjAPI-2
C4
SjAPI-2
+C4
C4
C4
Pepetde
